# Molecular surveillance of *Pfcrt* and *k13* propeller polymorphisms of imported *Plasmodium falciparum* cases to Zhejiang Province, China between 2016 and 2018

**DOI:** 10.1186/s12936-020-3140-0

**Published:** 2020-02-04

**Authors:** Xiaoxiao Wang, Wei Ruan, Shuisen Zhou, Fang Huang, Qiaoyi Lu, Xinyu Feng, He Yan

**Affiliations:** 1 National Institute of Parasitic Diseases, Chinese Center for Disease Control and Prevention, Key Laboratory of Parasite and Vector Biology, Schistosomiasis and Filariasis, MOH, and WHO Collaborating Centre for Malaria, Shanghai, People’s Republic of China; 2 Zhejiang Provincial Center for Disease Control and Prevention, Zhejiang, People’s Republic of China

**Keywords:** Drug resistance, *k13* propeller, Malaria, *Pfcrt*

## Abstract

**Background:**

Resistance to anti-malarial drugs hinders malaria elimination. Monitoring the molecular markers of drug resistance helps improve malaria treatment policies. This study aimed to assess the distribution of molecular markers of imported *Plasmodium falciparum* infections.

**Methods:**

In total, 485 *P. falciparum* cases imported from Africa, Southeast Asia, and Oceania into Zhejiang province, China, from 2016 to 2018 were investigated. Most were imported from Africa, and only a few cases originated in Asia and Oceania. Blood samples were collected from each patient. *Plasmodium falciparum* chloroquine resistance transporter (*Pfcrt*) at residues 72–76 and Kelch13-propeller (*k13*) were determined by nested PCR and DNA sequence.

**Results:**

Wild-type *Pfcrt* at residues 72–76 was predominant (72.61%), but mutant and mixed alleles were also detected, of which CVIET (22.72%) was the most common. Mutant *Pfcrt* haplotypes were more frequent in patients from West Africa (26.92%), North Africa (25%), and Central Africa (21.93%). The number of cases of *P. falciparum* infections was small in Southeast Asia and Oceania, and these cases involved *Pfcrt* mutant type. For the *k13* propeller gene, 26 samples presented 19 different point mutations, including eight nonsynonymous mutations (P441S, D464E, K503E, R561H, A578S, R622I, V650F, N694K). In addition, R561H, one of the validated SNPs in *k13*, was detected in one patient from Myanmar and one patient from Rwanda. A578S, although common in Africa, was found in only one patient from Cameroon. R622I was detected in one sample from Mozambique and one sample from Somalia. The genetic diversity of *k13* was low in most regions of Africa and purifying selection was suggested by Tajima’s D test.

**Conclusions:**

The frequency and spatial distributions of *Pfcrt* and *k13* mutations associated with drug resistance were determined. Wild-type *Pfcrt* was dominant in Africa. Among *k13* mutations correlated with delayed parasite clearance, only the R561H mutation was found in one case from Rwanda in Africa. Both *Pfcrt* and *k13* mutations were detected in patients from Southeast Asia and Oceania. These findings provide insights into the molecular epidemiological profile of drug resistance markers in the study region.

## Background

Malaria is a significant public health problem because of its worldwide distribution and high mortality. It was estimated that there were 219 million cases of malaria globally in 2017, mostly in 15 sub-Saharan African countries and India, representing approximately 80% of the global malaria burden [1]. Of the five malaria species, *Plasmodium falciparum* caused the most malaria incidence worldwide, accounting for 99.7% of estimated malaria cases in the World Health Organization (WHO) African region and 62.8% in Southeast Asia in 2017, and is the causative agent of the most severe forms of the disease [1].

Effective treatment is critical. Since the 1940s, multiple anti-malarial drugs have been developed and used to treat malaria parasites, including chloroquine (CQ), mefloquine, quinine, pyrimethamine, and sulfadoxine. However, the widespread use of these drugs promotes drug resistance, especially chloroquine resistance (CQR). Resistance to CQ occurred in Southeast Asia, South America, and the Western Pacific region in the late 1950s and rapidly spread to malaria-endemic areas, including Africa [2, 3]. Mutations in the chloroquine resistance transporter (*Pfcrt*) located on the *P. falciparum* digestive vacuole membrane were responsible for CQ treatment failure [4, 5]. Amino acid polymorphisms at PfCRT amino acid residues 72–76 were observed in CQR field isolates, whereas CVMNK haplotypes at PfCRT residues 72–76 were regarded as chloroquine sensitive (CQS) [6, 7]. Other studies revealed that *Pfcrt* K76T variants could affect parasite fitness, increase the rate of gametocyte production, and alter the susceptibility to artemisinin-based combination therapy (ACT) [3, 8–10]. These results highlight the need to monitor the molecular evolution of *Pfcrt*.

In view of *P. falciparum* multidrug resistance, the WHO recommended ACT as the first-line treatment for uncomplicated *P. falciparum* malaria in 2006 [11]. However, the detection of artemisinin-resistant *P. falciparum* in western Cambodia and the border between Cambodia and Thailand in 2008 was a drawback to malaria elimination [12, 13]. Over 200 nonsynonymous *P. falciparum* Kelch13 (*k13*) mutations have been reported to date, of which nine variants (F446I, N458Y, M476I, Y493H, R539T, I543T, P553L, R561H, and C580Y) were correlated with slow parasite clearance and reduced in vitro drug sensitivity, and over 20 *k13* mutations are considered candidates or associated markers [14]. *k13* mutations were detected predominantly in the Greater Mekong subregion(GMS) [15]. *k13* mutations are rare in Africa, and their profile is highly heterogeneous [16]. The prevalence of nonsynonymous *k13* mutations is low in approximately 50% of African countries [15]. Nevertheless, the increase in drug resistance in Africa could hamper malaria control considering its high morbidity and mortality. Therefore, monitoring mutations associated with artemisinin resistance via delayed parasite clearance globally, but especially in Africa, is critical.

Zhejiang province, located in eastern China, was considered malaria-free in 2018. No indigenous malaria infections have been reported in Zhejiang province since 2011. However, approximately 200 cases are imported every year, especially *P. falciparum* malaria from Africa. In this study, samples were collected from *P. falciparum* cases imported into Zhejiang Province, China, between 2016 and 2018, and molecular surveillance of *Pfcrt* and *k13* was performed to determine the emergence and spread of drug resistance in the countries of origin.

## Methods

### Sample collection and DNA extraction

Zhejiang province located in the Yangtze River Delta region and has many migrant workers travelling from Africa and Southeast Asia each year. Since 2012, all reported cases are imported. Microscope examination and PCR targeting the DNA of the *P. falciparum* multicopy 18S ribosomal RNA gene were employed to confirm suspected infections.

In this study, 485 cases of *P. falciparum* imported into Zhejiang Province, China between January 2016 and December 2018 were investigated. Venous blood was obtained from each patient. In total, 485 whole blood samples were collected. All blood samples were stored at − 80 °C until use. Parasite genomic DNA was extracted from 200 μL of blood using the QIAamp DNA Mini kit (QIAGEN Inc., Germany) following the manufacturer’s instructions.

### DNA amplification and sequencing

The *k13* and *Pfcrt* genes were amplified by nested PCR, as previously described [17, 18]. The primers for PCR were described in previous study and they are listed in Additional file 1: Table S1 [17, 18]. Proof-reading polymerase was used in each reaction to prevent amplification errors. The proof-reading polymerase was contained in the High Fidelity PCR Master Mix (item number B639292), which was supplied by Sangon Biotech Co., Ltd. (Shanghai, China). The amplification conditions in both PCR rounds were as follows: one cycle at 95 °C for 2 min, followed by 30 cycles at 95 °C for 2 s, 60 °C for 90 s, and 72 °C for 90 s, and one extension cycle at 72 °C for 10 min. PCR products were sequenced by Sangon Biotech Co., Ltd. (Shanghai, China).

### Data analysis

Mega version 7.0.26 (https://www.megasoftware.net/) was used to align amplicon sequences to 3D7 reference strain sequences retrieved from the NCBI database. A database was constructed using Microsoft Excel 2017, and statistical analysis was performed with SPSS Statistics for Windows version 21.0 (IBM Corp., Armonk, NY, USA). P-distance, the proportion of nucleotide sites at which two sequences being compared are different, was obtained by dividing the number of nucleotide differences by the total number of nucleotides compared. It was estimated by Mega version 7.0.26. Nucleotide diversity (Jukes and Cantor) (Pi (JC)), the average number of nucleotide substitutions per site between two sequences, was obtained using the Jukes and Cantor correction. Tajima’s D test was used as a neutrality test. Number of segregating sites (S), haplotype diversity (Hd), Pi (JC), average number of nucleotide differences (k) and Tajima’s D test were performed using DnaSP 6.12.03. Pearson Chi-square test was used for statistical analysis. Variables with a *P* value smaller than 0.05 were considered statistically significant.

## Results

### General information

**Fig. 1:**
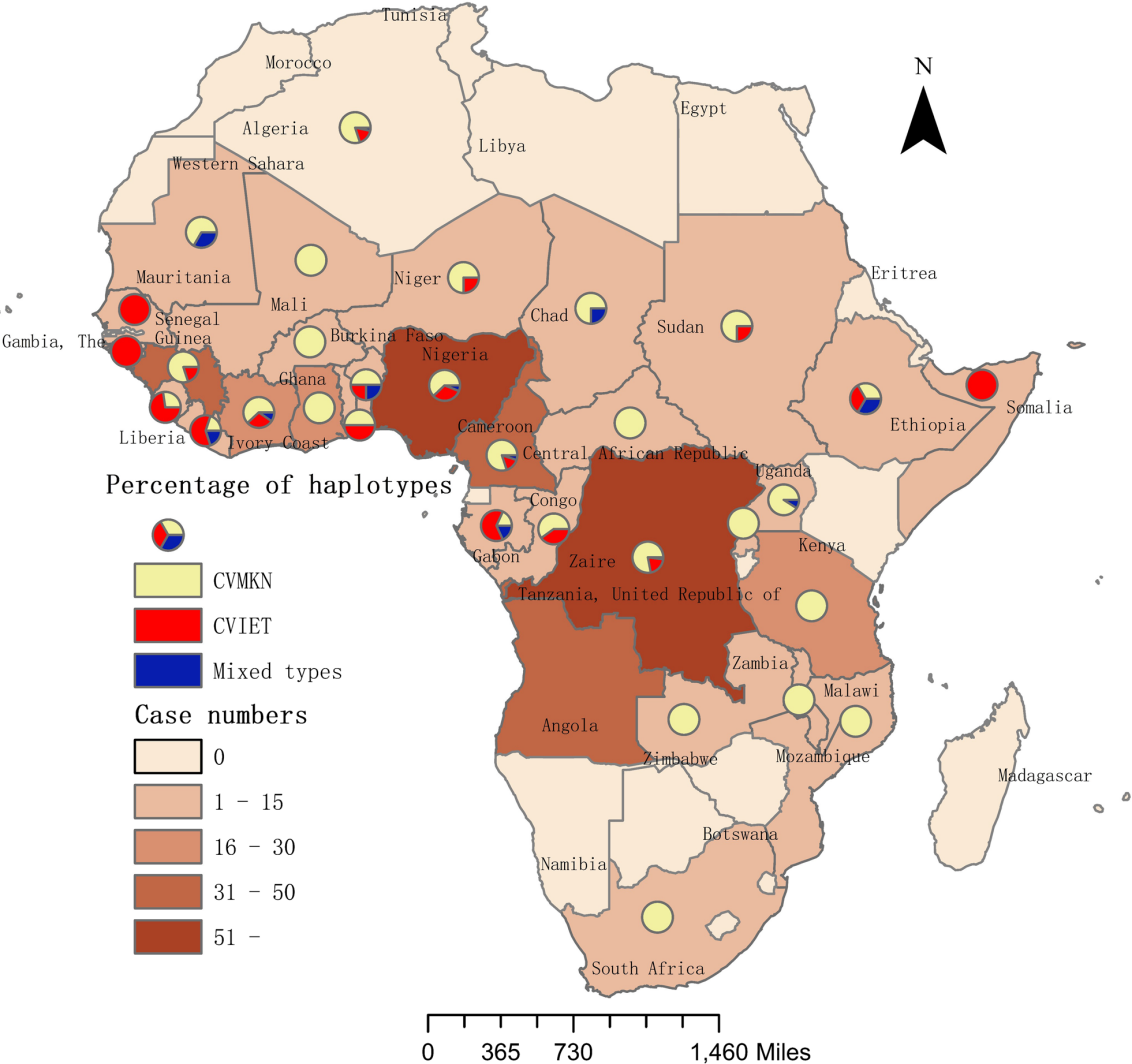
The number of imported *P. falciparum* cases from Africa and percentage of haplotypes of *Pfcrt*

A total of 485 *P. falciparum* cases imported from 37 countries from 2016 to 2018 were included in this study. Most cases involved migrant workers returning from Africa. Blood samples were collected from each patient, and 192, 169, and 124 samples were collected in 2016, 2017, and 2018, respectively (Additional file 1: Table S2). The median (range) age of the study population was 42 (9–69) years. A total of 426 out of 485 patients were men. Most cases were imported from West Africa (54.43%, 264/485), Central Africa (24.54%,119/485), and South Africa (12.16%, 59/485) (Fig. 1). Only a few cases originated in East Africa, North Africa, Philippines, Myanmar and Papua New Guinea, accounting for 6.80% (33/485), 0.82% (4/485), 0.21% (1/485), 0.21% (1/485) and 0.82% (4/485), respectively.

### *Pfcrt* mutations

**Fig. 2:**
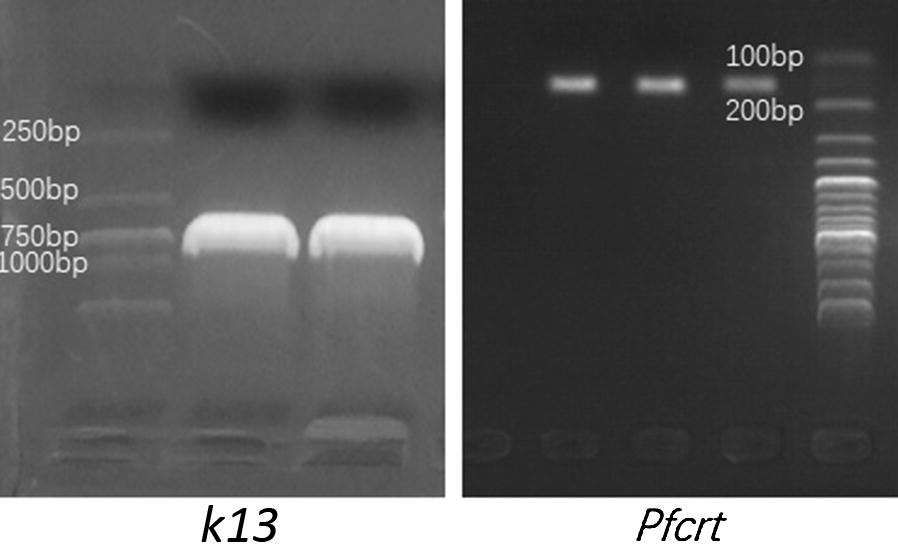
Electropherograms of *k13* and *Pfcrt* genotypes detected in imported *P. falciparum* cases

The amplified 145 bp fragment of *Pfcrt* encoded amino acid residues 72 to 76. Of the 485 samples, 471 were sequenced successfully, whereas 14 isolates [DR Congo (n = 2), Gabon (n = 2), Congo (n = 1), Mozambique (n = 1), Angola (n = 2), Malawi (n = 1), Republic of South Africa (n = 1), Guinea (n = 1), Nigeria (n = 3)] were not sequenced successfully because of the poor quality of DNA (Table 1). Wild-type *Pfcrt* alleles (CVMNK), mutant *Pfcrt* alleles (CVIET, SVMNT) and mixed *Pfcrt* alleles (CVM/I N/E/D/K K/T) were detected, and the most prevalent alleles were wild-type CVMNK (72.61%, 342/471) and mutant (22.72%, 107/471). Of the mixed haplotypes, the nucleotide sequences corresponding to residues 74–76 were ATG/T, A/GAA/T, and AA/CA, and could encode different amino acid sequences (CVM/I N/E/D/K K/T) (Table 1, Fig. 2).

**Table 1:** Prevalence of *Pfcrt* genotypes between 2016 and 2018

Region	Country	No	Prevalence (%)		
			Wild type (CVMNK)	Mutation type (CVIET/SVMNT)	Mixed type (CVM/I N/E/D/K K/T)
Central Africa		114	83 (72.81)	25 (21.93)	6 (5.26)
	DR Congo	50	39 (78.00)	10 (20.00)	1 (2.00)
	Cameroon	40	32 (80.00)	6 (15.00)	2 (5.00)
	Central African Republic	4	4 (100.00)	0 (0)	0 (0)
	Chad	4	3 (75.00)	0(0)	1 (25.00)
	Congo	5	3 (60.00)	2 (40.00)	0 (0)
	Gabon	11	2 (18.18)	7 (63.64)	2 (18.18)
East Africa		33	29 (87.88)	2 (6.06)	2 (6.06)
	Ethiopia	3	1 (33.33)	1 (33.33)	1 (33.33)
	Somalia	1	0 (0)	1 (100.00)	0 (0)
	Rwanda	1	1 (100.00)	0 (0)	0 (0)
	Tanzania	17	17 (100.00)	0 (0)	0 (0)
	Uganda	11	10 (90.91)	0 (0)	1 (9.09)
North Africa		4	3 (75.00)	1 (25.00)	0(0)
	Sudan	4	3 (75.00)	1 (25.00)	0(0)
South Africa		54	48 (88.89)	5 (9.26)	1 (1.85)
	Mozambique	11	11 (100.00)	0 (0)	0 (0)
	Angola	30	24 (80.00)	5 (16.67)	1 (3.33)
	Malawi	5	5 (100.00)	0 (0)	0 (0)
	Republic of south Africa	4	4 (100.00)	0 (0)	0 (0)
	Zambia	4	4 (100.00)	0 (0)	0 (0)
West Africa		260	177 (68.08)	70 (26.92)	13 (5.00)
	Republic of Niger	10	8 (80.00)	2 (20.00)	0 (0)
	Ghana	27	27 (100.00)	0 (0)	0 (0)
	Benin	8	4 (50.00)	2 (25.00)	2 (25.00)
	Burkina Faso	2	2 (100.00)	0 (0)	0 (0)
	Côte d’Ivoire	21	13 (61.90)	6 (28.57)	2 (9.52)
	Guinea	48	39 (81.25)	9 (18.75)	0 (0)
	Guinea-Bissau	2	1 (50.00)	1 (50.00)	0 (0)
	Liberia	5	1 (20.00)	3 (60.00)	1 (20.00)
	Mali	2	2 (100.00)	0 (0)	0 (0)
	Mauritania	3	2 (66.67)	0 (0)	1 (33.33)
	Nigeria	118	74 (62.71)	37 (31.36)	7 (5.93)
	Sierra Leone	11	3 (27.27)	8 (72.73)	0 (0)
	Senegal	1	0 (0)	1 (100.00)	0 (0)
	Togo	2	1 (50.00)	1 (50.00)	0 (0)
Southeast Asia		2	1 (50.00)	1 (50.00)	0 (0)
	Philippines	1	0 (0)	1 (100.00)	0 (0)
	Myanmar	1	1 (100.0)	0 (0)	0 (0)
Oceania		4	1 (25.00)	3 (75.00)	0 (0)
	Papua New Guinea	4	1 (25.00)	3^a^ (75.00)	0 (0)
Total		471	342 (72.61)	107 (22.72)	22 (4.67)

^a^The 3 samples harboured SVMNT haplotypes

### Spatial distribution of *Pfcrt* mutations

*Pfcrt* mutations were more common in patients from West Africa (26.92%, 70/260), North Africa (25.00%, 1/4) and Central Africa (21.93%, 25/114). The smallest percentage of *Pfcrt* mutations was found in East Africa (6.06%, 2/33). The difference in the prevalence of *Pfcrt* mutations between West, Central, North, East, and South Africa was statistically significant (χ^2^ = 14.165, *P *< 0.05). The spatial distribution of these mutations was highly heterogeneous. The percentage of wild-type, mutant, and mixed haplotypes varied greatly between countries. Although the number of cases originating in Asia and Oceania was small, these cases had a high rate of the mutant type. One out of two patients returning from Asia presented mutant alleles CVIET and three out of four patients returning from Papua New Guinea harbored SVMNT haplotypes (Table 1, Fig. 1).

### *k13* propeller mutations

The nested PCR yielded an 849-bp amplification product. Among the 485 samples, 437 were successfully amplified and sequenced (Central Africa:106, East Africa: 21, North Africa: 4, South Africa: 53, West Africa: 247, Southeast Asia: 2, Oceania: 4). Twenty-six samples presented 19 different point mutations, including eight nonsynonymous mutations (P441S, D464E, K503E, R561H, A578S, R622I, V650F, N694K) (Table 2). DNA sequences with the 19 point mutations were submitted to the NCBI database under GenBank accession numbers MN586239 to MN586257. It is worth noting that an R561H mutation, one of the nine validated molecular markers associated with for artemisinin resistance via delayed parasite clearance, was detected in two patients, one each from Myanmar and Rwanda. Moreover, two samples harboring a P441S mutation contained either mutant or mixed *Pfcrt* haplotypes (Table 2). The A578S substitution, although common in Africa, was only found in one patient returning from Cameroon, and K503E was identified in another sample. In addition, R622I was detected in two samples, one each from Mozambique and Somalia. V650F, N694K, and D464E substitutions were present in parasite strains from Nigeria, Côte d’Ivoire, Angola, and Guinea, respectively.

**Table 2:** Mutant types observed in the *k13* of imported *P. falciparum* infections

Codon position	Amino acid reference	Nucleotide reference	Amino acid mutation	Nucleotide mutation	Location and year (No.)
441	P	ACC	S	ATC	Nigeria 2017 (1)^a^, 2018 (1)^b^
464	D	GAT	E	GAA	Guinea 2018 (1)
469	C	TGC	C	TGT	Nigeria 2017 (1)
471	R	CGT	R	CGC	Angola 2016 (1)
478	T	ACC	T	ACG	Benin 2017 (1)
492	L	TTA	L	TTG	Ghana 2016 (1)
493	Y	TAC	Y	TAT	Liberia 2017 (1), Côte d’Ivoire 2018 (1)
496	G	GGT	G	GGC	Ghana 2018 (1)
503	K	AAG	E	GAG	Cameroon 2018 (1)
535	T	ACG	T	ACA	Côte d’Ivoire 2017 (1)
561	R	CGT	H	CAT	Rwanda 2017 (1), Myanmar 2017 (1)
578	A	GCT	S	TCT	Cameroon 2016 (1)
589	V	GTC	V	GTG	Nigeria 2016 (2)
622	R	AGA	I	ATA	Mozambique 2016 (1), Somalia 2016 (1)
638	G	GGA	G	GGG	Republic of Niger 2017 (1)
650	V	GTT	F	TTT	Nigeria 2016 (1)
664	N	AAT	N	AAC	Nigeria 2017 (1)
676	A	GCC	A	GCT	Nigeria 2016 (1), Guinea 2016 (1)
694	N	AAT	K	AAG	Côte d’Ivoire 2017 (1), Angola 2017 (1)

*No.* number of isolates

^a^Isolate with mutant type of *Pfcrt*

^b^Isolate with mixed type of *Pfcrt*

### Genetic diversity of *k13*

The genetic diversity of the *k13* gene represented by the P-distance, S, Hd, Pi, and k values was low in the imported samples (Table 3). Similarly, only 15 haplotypes were observed in isolates imported from West Africa, with a haplotype diversity of 0.141. The patients from East Africa only had two haplotypes, but the haplotype diversity, nucleotide diversity, and average number of nucleotides were the highest compared with those from other areas. After combining all the samples, Tajima’s D significantly deviated from neutrality. However, when the samples from the different regions were analysed separately, the negative Tajima’s D on *k13* was only evident in the isolates from West Africa. The Tajima’s D values for the samples from South Africa, Central Africa, and East Africa were not statistically significant, but they exhibited a similar trend to that obtained for the samples from West Africa.

**Table 3:** Genetic diversity of *k13* gene from imported *P. falciparum* infections

Region	n	P-distance	S	Hd (SD)	Pi(JC)	k	Tajima’s D	*p*-value^△^
West Africa	247	0.000	15	0.141 (0.030)	0.0002	0.162	− 2.32586	< 0.01
South Africa	53	0.000	3	0.111 (0.059)	0.00014	0.113	− 1.68986	> 0.05
Central Africa	106	0.000	2	0.038 (0.026)	0.00005	0.038	− 1.37037	> 0.10
East Africa	21	0.000	2	0.186 (0.110)	0.00024	0.190	− 1.51414	> 0.10
Total^a^	427	0.000	20	0.114 (0.021)	0.00016	0.126	− 2.38774	< 0.01

*S* number of segregating sites, *Hd* haplotype diversity, *Pi (JC)* nucleotide diversity (Jukes and Cantor), *k* average number of nucleotide differences

^a^Sequences from North Africa, Southeast Asia and Oceania were not included due to their limited sample size or number of mutants

^△^*p*-value for Tajima’s D

## Discussion

As one of the most important approaches to combat malaria, chemotherapy is paramount to treat *Plasmodium* and interrupt malaria transmission. However, effective anti-malarial drug policies were followed by drug resistance, which temporarily interrupted malaria elimination campaigns. Drug resistance has posed a significant threat to global malaria control strategies and raised international concern, especially in areas most strongly affected by the disease [19]. Most malaria cases originated in the WHO African region, accounting for approximately 92% of all cases and 93% deaths [1]. Four countries in Africa represented almost 50% of all malaria cases worldwide: Nigeria (25%), The Democratic Republic of Congo (11%), Mozambique (5%), and Uganda (4%) [1]. Furthermore, African countries spread the infection to other countries, including France (> 2500), China (> 2000), UK (approximately 1500), and Germany (> 500) in 2017 [1, 20]. Zhejiang province, located in southeast China, had several cases of *P. falciparum* malaria imported from Africa in recent years [21, 22]. The current study evaluated drug resistance markers of *P. falciparum* infections predominantly from Africa using molecular assays to advance drug policies against malaria.

Chloroquine, an easy-to-use and affordable first-line antimalarial agent, is comprehensively used globally to treat *P. falciparum* and *Plasmodium vivax*. Nevertheless, this drug was withdrawn from most endemic countries due to the high levels of resistance, which has resulted in a two- or threefold increase in malaria deaths and hospital admission for severe malaria in various African countries [19, 23]. Over ten multi-mutations sharing the common K76T substitution have been detected and are associated with CQR in field and laboratory strains of *P. falciparum* [4, 24, 25]. Five major haplotypes at *Pfcrt* residues 72–76 (CVIET, SVMNT, SVIET, CVMNT, and CVTNT) were related to CQR, and CVIET and SVMNT were regarded as the most resistant haplotypes [7, 26–28]. CVIET is predominant in Africa, whereas SVMNT is more common in South America [27]. Our study confirmed that CVIET was the most common mutation type in infections imported from Africa, similar to previous studies [26, 28]. The high frequency of SVMNT in Papua New Guinea was also consistent with other studies [29]. It was demonstrated that the spatial distribution of mutant alleles was mainly related to local drug policy. The increase in the prevalence of SVMNT isolates in Tanzania from 0% (0/156) in 2003 to 3.68% (6/163) in 2004 was due to selective pressure for amodiaquine resistance [27]. Therefore, it was postulated that CVIET might be displaced by SVMNT in African regions where amodiaquine was increasingly used [27, 30]. However, our study refuted this hypothesis by showing the absence of SVMNT double mutants in Tanzania and other African countries. The disagreement in the results may be due to differences in sample size and survey sites. The present results indicate that parasites with CVIET are still a major threat in Africa.

With the cessation of CQ use between 1998 and 2008, parasite populations with wild-type CVMNK returned progressively, demonstrated by the increased frequency of this haplotype in Africa [30–33]. For instance, the frequency of a CQS genotype increased from 28.0% in 2003 to 53.7% in 2012 after disuse of CQ for 9 years in Cameroon [34]. This hypothesis is supported in our study by the consistent detection of CVMNK (72.61%) in imported malaria cases and by another study wherein most *P. falciparum* isolates from Africa harbored wild-type alleles [33]. A possible explanation is that CVIET mutants were not fit enough to evolve into wide-type strains without selective pressure [30]. These results help understand the dynamics of major *Pfcrt* haplotypes in Africa and enrich the evidence for drug policy.

K13 propeller polymorphisms in *P. falciparum* have been widely studied since it was first described in GMS [12]. Molecular markers for *k13* have been identified and can help elucidate artemisinin delayed parasite clearance [15]. In this study, 19 alleles were identified, and eight (P441S, D464E, K503E, R561H, A578S, R622I, V650F, and N694K) were nonsynonymous. Similarly with previous literatures, the propeller domain of the *k13* gene showed a limited diversity of alleles in Africa [18, 35, 36]. Of note is that one of the validated *k13* resistance mutations—R561H—was detected in two patients, one each from Rwanda and Myanmar. R561H in Myanmar and western Thailand, although not the predominantly popular, was more prevalent than that in Africa [33, 37, 38]. The only infected patient from Myanmar was positive for R561H. Previous studies found the R561H allele in Congo. However, R561H might be the first time found from Rwanda [36, 37, 39]. In addition, mutation A578S identified in one isolate from Cameroon, was also found in other African countries, including Gabon, Uganda, Mali, Kenya and DR Congo [18, 39–41], but it was not associated with clinical or in vitro resistance to artemisinin according to previous studies [37, 42]. The study also found the uncommon *k13* mutation R622I in two cases, one in Mozambique and one in Somalia, and this mutation was initially reported in northwest Ethiopia at the border with Sudan [43]. A previous study showed that, of a total of over 14,000 screened samples from 59 countries in Africa, Asia, Oceania, and South America, only one sample from Zambia was positive for R622I [37]. Interestingly, one out of three patients from Ethiopia bearing R622I mutation showed day-3 positivity in Giemsa-stained smears [43]. Further clinical studies are required to investigate the role of R622I in acquired resistance to artemisinin.

In Nigeria, where nearly 25% of the samples collected in this study originated, seven samples exhibited six mutant types (two nonsynonymous types comprising P441S and V650F, and four nonsynonymous types comprising C469C, V589V, N664N, and A676A). Similarly, no validated or candidate amino acid mutations were detected in southwestern Nigeria according to recent studies [35, 44]. However, a low prevalence of single nucleotide polymorphisms (G665C, V666V, P553P, V510V, A578S, D464N, and Q613H) were also reported, although they differed from the results obtained in the current study [35, 44]. In general, these results indicate that the profiles of the molecular markers conferring artemisinin delayed clearance were still optimistic with respect to the public health situation in terms of malaria in Nigeria.

The molecular genetic analyses conducted in this study showed that the haplotype diversity of the samples imported Africa was relatively low. Similarly, haplotype diversity of *P. falciparum* isolates in Congo, Ghana, Kenya and Tanzania in a previous study was 0.067, 0.123, 0.066 and 0.056, respectively [45]. Also, southwestern Nigeria reported Hd 0.080–0.157 in two states [35]. However, another research reported Hd 0.74 from African isolates [46]. The difference might result from heterogeneity of spatial distribution of samples. The current study also showed that the sequence diversity varied among the isolates from West, South, East, and Central Africa. The isolates from East Africa had the highest genetic diversity, whereas those from Central Africa had the lowest diversity. The discrepancy might have been related to the unequal sample sizes in different areas. Further investigation is required. Regarding the neutrality test, there was no consistent results. Tajima’s D value in this study was found to be negative and statistically significantly in terms of the total samples from Africa. Another research also reported similar trends from African isolates [46]. Nevertheless, negative Tajima’s D value without statistical significance was observed in Nigeria which indicated *k13* gene evolved under neutral model [35].

## Conclusions

In conclusion, this study determined the frequency and spatial distribution of *Pfcrt* and *k13* mutations associated with drug resistance in *P. falciparum* malaria cases imported into Zhejiang province. In Africa, wild-type *Pfcrt* was predominant, but detection of *k13* mutants was limited by the absence of genetically validated molecular markers of artemisinin delayed parasite clearance, except one case from Rwanda harbouring the R561H mutation. Both *Pfcrt* and *k13* mutations were detected in patients from Southeast Asia and Oceania. These results may guide efforts to make more rational and targeted drug policies to eliminate resistant malaria in the study region and demonstrated that molecular markers are an effective and convenient tool to improve the surveillance of drug resistance.

## Supplementary information

**Additional file 1: Table S1.** Primers for *Pfcrt* and K13 propeller genotyping assay. **Table S2.** Distribution of imported *P. falciparum* cases of Zhejiang province between 2016 and 2018.

## Data Availability

The datasets analysed in this study are available from the corresponding author on reasonable request.

## Abbreviations

*Pfcrt*: *Plasmodium falciparum* chloroquine resistance
CQ: Chloroquine
CQR: Chloroquine resistance
CQS: Chloroquine sensitive
*k13*: Kelch13
ACT: Artemisinin-based combination therapy
GMS: Greater Mekong subregion
