# Supplementary material for: Molecular surveillance of Pfcrt and k13 propeller polymorphisms of imported Plasmodium falciparum cases to Zhejiang Province, China between 2016 and 2018
Source: Malar J. 2020 Feb 4;19:59. doi: 10.1186/s12936-020-3140-0 (PMC7001319; doi:10.1186/s12936-020-3140-0)
Supplement: Supplementary file 1 — Additional file 1: Table S1. Primers for Pfcrt and K13 propeller genotyping assay. Table S2. Distribution of imported P. falciparum cases of Zhejiang province between 2016 and 2018. [file 12936_2020_3140_MOESM1_ESM.docx]

Table S1 Primers for *Pfcrt* and K13 propeller genotyping assay

| Gene | Round | Primer name | Sequence (5’→3’) | Reference |
| --- | --- | --- | --- | --- |
| K13 | Primary | K13-1-F | CGGATGACCAAATCTGGGA | [18] |
|  |  | K13-1-R | GGGAATCTGGTGGTAACAGC |  |
|  | Secondary | K13-2-F | GCCAAGCTGCCATTCATTTG |  |
|  |  | K13-2-R | GCCTTGTTGAAAGAAGCAGA |  |
| *Pfcrt* | Primary | *Pfcrt*-1-F | CCCTTGTCGACCTTAACAGATG | [17] |
|  |  | *Pfcrt*-1-R | AAAATGACTGAACAGGCATCTAAC |  |
|  | Secondary | *Pfcrt*-2-F | TCTTGGTAAATGTGCTCATGTG |  |
|  |  | *Pfcrt*-2-R | AAAGTTGTGAGTTTCGGATGTT |  |

Table S2 Distribution of imported *P.falciparum* cases of Zhejiang province between 2016 and 2018

| Region | Country | Year | | | Subtotal |
| --- | --- | --- | --- | --- | --- |
|  |  | 2016 | 2017 | 2018 |  |
| Central Africa |  | 56 | 39 | 24 | 119 |
|  | DR Congo | 34 | 10 | 8 | 52 |
|  | Cameroon | 15 | 14 | 11 | 40 |
|  | Central African Republic | 2 | 2 | 0 | 4 |
|  | chad | 1 | 2 | 1 | 4 |
|  | Congo | 2 | 3 | 1 | 6 |
|  | Gabon | 2 | 8 | 3 | 13 |
| East Africa |  | 12 | 13 | 8 | 33 |
|  | Ethiopia | 1 | 1 | 1 | 3 |
|  | Somalia | 1 | 0 | 0 | 1 |
|  | Rwanda | 0 | 1 | 0 | 1 |
|  | Tanzania | 7 | 6 | 4 | 17 |
|  | Uganda | 3 | 5 | 3 | 11 |
| North Africa |  | 1 | 0 | 3 | 4 |
|  | Sudan | 1 | 0 | 3 | 4 |
| South Africa |  | 32 | 19 | 8 | 59 |
|  | Mozambique | 7 | 4 | 1 | 12 |
|  | Angola | 18 | 9 | 5 | 32 |
|  | Malawi | 3 | 2 | 1 | 6 |
|  | Republic of south Africa | 2 | 3 | 0 | 5 |
|  | Zambia | 2 | 1 | 1 | 4 |
| West Africa |  | 89 | 94 | 81 | 262 |
|  | Republic of Niger | 3 | 5 | 2 | 10 |
|  | Ghana | 9 | 11 | 7 | 27 |
|  | Benin | 1 | 3 | 4 | 8 |
|  | Burkina Faso | 1 | 0 | 1 | 2 |
|  | Côte d'Ivoire | 5 | 9 | 7 | 21 |
|  | Guinea | 17 | 13 | 19 | 49 |
|  | Guinea-Bissau | 0 | 0 | 2 | 2 |
|  | Liberia | 1 | 3 | 1 | 5 |
|  | Mali | 2 | 0 | 0 | 2 |
|  | Mauritania | 2 | 1 | 0 | 3 |
|  | Nigeria | 46 | 45 | 30 | 121 |
|  | Sierra Leone | 1 | 4 | 6 | 11 |
|  | Senegal | 1 | 0 | 0 | 1 |
|  | Togo | 0 | 0 | 2 | 2 |
| Southeast Asia |  | 1 | 1 | 0 | 2 |
|  | Philippines | 1 | 0 | 0 | 1 |
|  | Myanmar | 0 | 1 | 0 | 1 |
| Oceania |  | 1 | 3 | 0 | 4 |
|  | Papua New Guinea | 1 | 3 | 0 | 4 |
| Total | | 192 | 169 | 124 | 485 |
